# Development of targeted drugs for diabetic retinopathy using Mendelian randomized pharmacogenomics

**DOI:** 10.3389/fendo.2025.1632691

**Published:** 2025-10-09

**Authors:** Guodan Liu, Miao Tian, Xinge Li, Xichen Wang, Songhao Zhang, Gali Bai, Xuyang Zhang

**Affiliations:** Department of Ophthalmology, The Fourth Affiliated Hospital of Harbin Medical University, Harbin, China

**Keywords:** diabetic retinopathy, drug target, Mendelian randomization, latest updated articles, network analysis

## Abstract

**Purpose:**

This study aims to utilize genetic instrumental variables - protein quantitative trait loci (pQTL), and through analysis methods such as Mendelian randomization (MR), systematically screen and validate druggable proteins that have a causal relationship with diabetic retinopathy (DR), and further explore related drug targets, providing genetic evidence and new directions for the drug development of this disease.

**Methods:**

The research was based on large-scale public databases to conduct two-sample Mendelian randomization (MR) analysis. Firstly, 511 encoded proteins were selected from the known 4,479 druggable genes as initial exposure factors, with the summary data of GWAS for diabetic retinopathy as the outcome. MR analysis was conducted using the inverse variance weighted (IVW) method and the Wald ratio method, and strict screening was performed through Bonferroni correction. For the significantly associated proteins, heterogeneity tests, pleiotropy tests, leave-one-out analysis, and Steiger directionality tests were further conducted to verify the robustness of the results. Additionally, summary MR (SMR) analysis and colocalization analysis (coloc) were used to confirm the reliability of the causal relationship. Finally, a protein-protein interaction (PPI) network was constructed using the STRING database, and potential targeted drugs were mined from the DrugBank and DSigDB databases.

**Results:**

A preliminary analysis identified 37 proteins with potential causal relationships to DR (p < 0.05). After more rigorous pQTL screening and multiple testing corrections, it was found that Noggin (NOG) protein has a significant negative causal relationship with the risk of DR (p.adjust < 0.05), meaning that higher NOG protein levels may reduce the risk of disease. All sensitivity analyses supported the robustness of this result (no heterogeneity, no pleiotropy), and SMR and colocalization analyses (PP.H4 > 0.8) further confirmed this causal association. PPI network analysis revealed that NOG interacts with 10 proteins (such as BMP2, BMP4, etc.). Drug mining identified DB01373 as a corresponding drug for BMP4, and through DSigDB analysis, progesterone and estradiol were found to be potential therapeutic compounds targeting the NOG network.

**Conclusions:**

Through comprehensive genetic analysis, this study identified the NOG protein as a novel potential protective drug target for DR. Its function may be achieved by regulating the BMP signaling pathway. The research findings not only provide a new perspective for understanding the pathogenesis of this disease but also recommend existing drugs such as progesterone and estradiol as potential therapies, which are worthy of further functional experiments and clinical studies for verification.

## 1 Introduction

Diabetic retinopathy (DR), a microvascular complication of diabetes affecting approximately one-third of diabetic patients, is a leading cause of blindness (1). The In1ternational Diabetes Federation estimated that by 2030, nearly 191 million individuals will suffer from some degree of DR, with many at risk for loss of vision. In the early stages of DR, hyperglycemia may lead to oxidative stress, neurodegenerative diseases, alterations of the retinal blood vessel wall, and blood rheology, leading to retinal ischemia and hypoxia, retinal vascular leakage, and neovascularization. In the terminal stage of diabetic retina, severe ischemia and hypoxia may cause neovascularization, vitreous hemorrhage, and even retinal detachment (2).At present, the gold standard for the examination of DR remains fundus angiography, but ultra-wide-angle fundus photography and artificial intelligence analysis are gradually being applied (3). Martínez-García I et al. previously published an article indicating that non-invasive skin autofluorescence (SAF) could potentially serve as an accessible, rapid, and straightforward alternative for the screening and early diagnosis of diabetic retinopathy (DR). Nevertheless, this examination method has not been clinically implemented to date. If this approach can be translated into clinical practice in the future, it will alleviate the suffering associated with invasive procedures for patients with diabetic retinopathy, thereby bringing them significant benefits (4). Current treatments, such as control of blood glucose, blood pressure, cholesterol, and other indicators; laser photocoagulation; anti-VEGF injections; and vitrectomy are limited by their invasiveness, potential side effects, and variable patient response (5, 6). For instance, retinal laser photocoagulation is an invasive treatment that does not lead to improvement in the patient’s vision. Anti-VEGF therapy and vitrectomy are associated with high costs and often necessitate repeated treatments. These limitations underscore the urgent need to identify novel therapeutic targets for safer and more effective interventions.

Identification of novel drugs to treat DR is complicated by its multifactorial pathophysiology; hyperglycemia-induced metabolic changes lead to retinal microvascular damage and inflammation. Proteomic studies have revealed alterations in several proteins associated with DR progression (7). Furthermore, genetic factors contribute to individual susceptibility and disease severity. Genome-wide association studies (GWAS) have identified multiple loci associated with DR, but the causal relationships between these genetic polymorphisms and the disease remain largely unexplored (8).

Adding to the challenges in novel DR drug identification, traditional statistical methods for inferring causality from observational data are prone to confounding biases. Mendelian randomization (MR), which involves using genetic variants as instrumental variables for modifiable exposures, offers an alternative approach that mitigates these biases owing to the random assortment of alleles at conception (8, 9). In complex diseases like DR, MR could provide robust evidence for causal inference when randomized controlled trials are not feasible or ethical.

In this study, we aimed to identify potential drug targets for DR by employing a comprehensive bioinformatics approach. We integrated druggable gene selection, protein quantitative trait locus (pQTL) analysis, two-sample MR analysis, summary-data-based MR (SMR), colocalization analyses, and drug target validation through protein-protein interaction (PPI) networks and pharmacological databases. On this basis, verification was also conducted through external datasets, immune infiltration, and laboratory experiments. By elucidating the causal relationship between proteins implicated in DR and the disease itself, we sought to identify novel therapeutic strategies that would lead to more effective treatments.

## 2 Results

### 2.1 Technology roadmap

**Figure 1:**
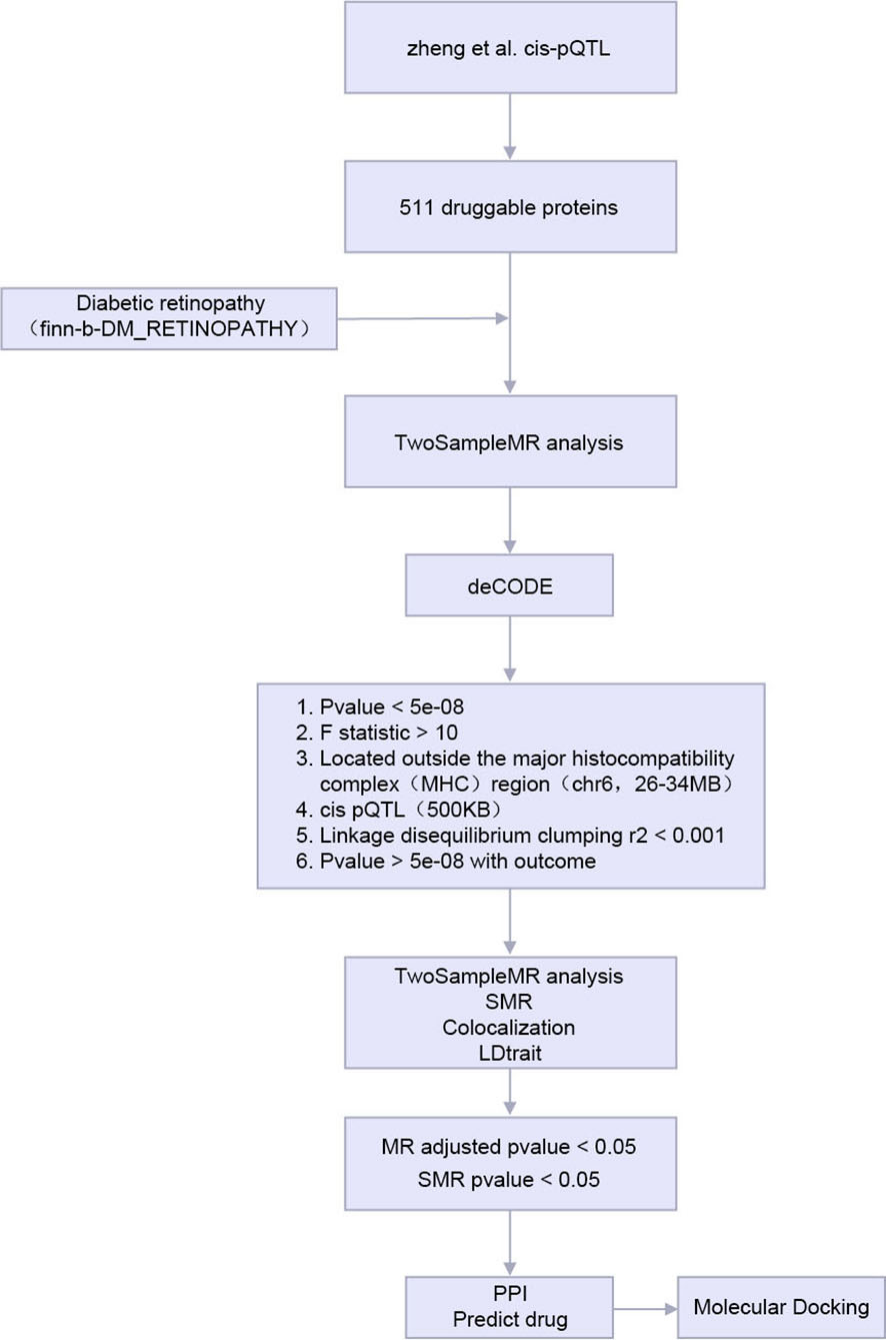
Technology roadmap.

MR, Mendelian Randomization; SMR, the Summary - data - -based Mendelian Randomization. PPI, Protein-protein interaction; pQTL, protein Quantitative Trait Locus.

The analysis flow of this study is shown in **Figure 1**.

### 2.2 MR analysis of pharmaceutically available proteins

We first intersected the 734 proteins studied by Zheng et al. with 4479 proteins encoded by druggable genes to obtain 511 proteins encoded by druggable genes. Then, we performed two-sample MR analysis of the 511 proteins and DR by using two-sample MR. We next performed initial screening using p.adjust < 0.05 for significant causal filters. A total of 37 proteins are shown in **Table 1** as a result of the analysis of causal relationships between proteins and DR.

**Table 1:** Mendelian randomization causal effect estimates of protein in the pathogenesis of diabetic retinopathy.

exposure	outcome	nsnp	b	se	OR (95% CI)	P value	method
ADAM23	Diabetic retinopathy	1	0.080536913	0.030779912	0.92 (0.87, 0.98)	8.88 e-03	Wald ratio
CFH	Diabetic retinopathy	1	0.21691678	0.03574352	0.80 (0.75, 0.86)	1.29 e-09	Wald ratio
PLA2G2A	Diabetic retinopathy	1	0.029691328	0.014306712	0.97 (0.94, 1.00)	3.80 e-02	Wald ratio
LY9	Diabetic retinopathy	1	0.063503475	0.031152648	1.07 (1.00, 1.13)	4.15 e-02	Wald ratio
GSTA1	Diabetic retinopathy	1	0.08082752	0.031031994	1.08 (1.02, 1.15)	9.20 e-03	Wald ratio
WFIKKN2	Diabetic retinopathy	1	0.044083882	0.020970458	1.05 (1.00, 1.09)	3.55 e-02	Wald ratio
COL18A1	Diabetic retinopathy	1	0.215060241	0.081927711	1.24 (1.06, 1.46)	8.66 e-03	Wald ratio
GP1BA	Diabetic retinopathy	1	0.138677233	0.064614447	1.15 (1.01, 1.30)	3.19 e-02	Wald ratio
CHL1	Diabetic retinopathy	1	0.126801153	0.063400576	0.88 (0.78, 1.00)	4.55 e-02	Wald ratio
CRTAM	Diabetic retinopathy	1	0.119915254	0.055508475	0.89 (0.80, 0.99)	3.07 e-02	Wald ratio
TGFB1	Diabetic retinopathy	1	0.114671815	0.054440154	1.12 (1.01, 1.25)	3.52 e-02	Wald ratio
HSPB1	Diabetic retinopathy	1	0.089166667	0.043333333	0.91 (0.84, 1.00)	3.96 e-02	Wald ratio
F13B	Diabetic retinopathy	1	0.097074954	0.027787934	1.10 (1.04, 1.16)	4.77 e-04	Wald ratio
CTSH	Diabetic retinopathy	1	0.043330427	0.016826504	1.04 (1.01, 1.08)	1.00 e-02	Wald ratio
COL6A1	Diabetic retinopathy	1	0.170524327	0.060935286	0.84 (0.75, 0.95)	5.13 e-03	Wald ratio
RNASE3	Diabetic retinopathy	1	0.181775701	0.076168224	1.20 (1.03, 1.39)	1.70 e-02	Wald ratio
CD59	Diabetic retinopathy	1	0.08480663	0.040331492	1.09 (1.01, 1.18)	3.55 e-02	Wald ratio
CPM	Diabetic retinopathy	1	0.161377084	0.081764389	0.85 (0.72, 1.00)	4.84 e-02	Wald ratio
NQO1	Diabetic retinopathy	1	0.073086156	0.022005868	1.08 (1.03, 1.12)	8.96 e-04	Wald ratio
IL7R	Diabetic retinopathy	1	0.073446328	0.030838041	1.08 (1.01, 1.14)	1.72 e-02	Wald ratio
PAM	Diabetic retinopathy	1	0.093053173	0.028945111	0.91 (0.86, 0.96)	1.31 e-03	Wald ratio
FGFR3	Diabetic retinopathy	1	0.175706215	0.081920904	0.84 (0.71, 0.98)	3.20 e-02	Wald ratio
CST5	Diabetic retinopathy	1	0.052675585	0.023968785	1.05 (1.01, 1.10)	2.80 e-02	Wald ratio
DUT	Diabetic retinopathy	1	0.166076696	0.078761062	0.85 (0.73, 0.99)	3.50 e-02	Wald ratio
PPT1	Diabetic retinopathy	1	0.046978309	0.022851177	1.05 (1.00, 1.10)	3.98 e-02	Wald ratio
CFHR1	Diabetic retinopathy	1	0.037606178	0.017142857	1.04 (1.00, 1.07)	2.83 e-02	Wald ratio
BST1	Diabetic retinopathy	1	0.034295124	0.013245507	0.97 (0.94, 0.99)	9.62 e-03	Wald ratio
NOG	Diabetic retinopathy	1	0.174306735	0.049074299	0.84 (0.76, 0.92)	3.82 e-04	Wald ratio
LAMC2	Diabetic retinopathy	1	0.045166531	0.021933387	1.05 (1.00, 1.09)	3.95 e-02	Wald ratio
NID2	Diabetic retinopathy	1	0.064941654	0.032724505	1.07 (1.00, 1.14)	4.72 e-02	Wald ratio
CHRDL2	Diabetic retinopathy	1	0.150490731	0.049384639	0.86 (0.78, 0.95)	2.31 e-03	Wald ratio
NEGR1	Diabetic retinopathy	1	0.171621622	0.087162162	0.84 (0.71, 1.00)	4.90 e-02	Wald ratio
CLEC4C	Diabetic retinopathy	1	0.032105263	0.015052632	0.97 (0.94, 1.00)	3.29 e-02	Wald ratio
CFHR4	Diabetic retinopathy	1	0.102442748	0.021526718	1.11 (1.06, 1.16)	1.95 e-06	Wald ratio
RTN4R	Diabetic retinopathy	1	0.120354488	0.043453402	1.13 (1.04, 1.23)	5.61 e-03	Wald ratio
CPA4	Diabetic retinopathy	1	0.03139475	0.011408475	0.97 (0.95, 0.99)	5.93 e-03	Wald ratio
ENPP5	Diabetic retinopathy	1	0.040805981	0.018121911	1.04 (1.01, 1.08)	2.43 e-02	Wald ratio

SNP, single nucleotide polymorphism; OR, odds ratio; CI, confidence interval.

**Figure 2:**
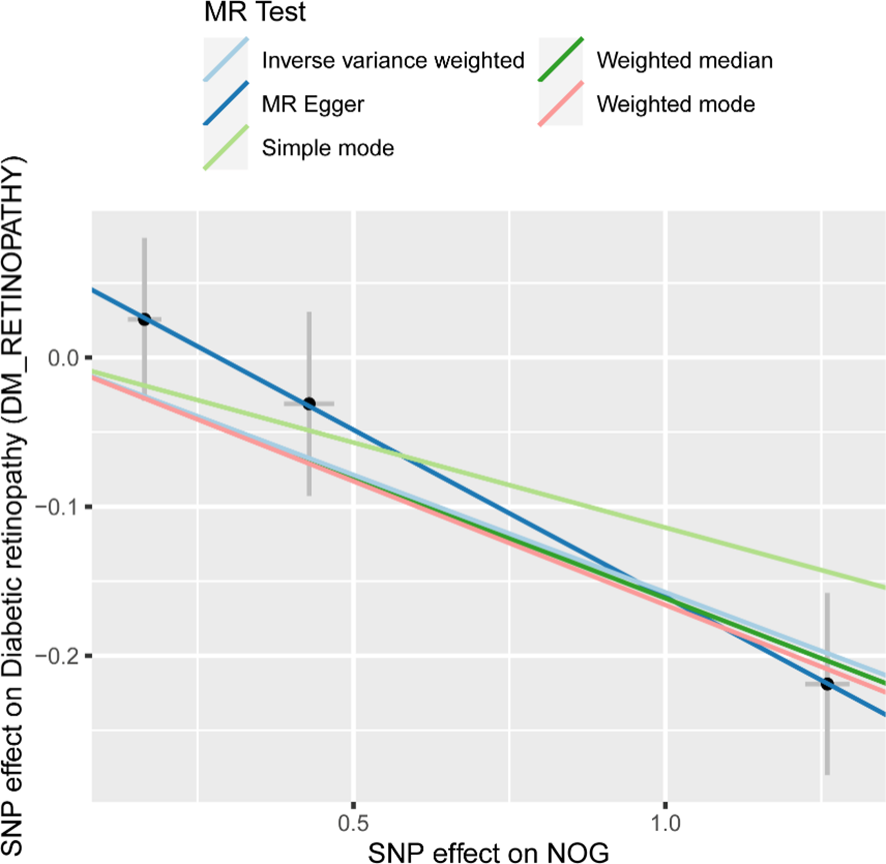
Scatter plots of effect estimates for different models of MR analysis of proteins for DR. MR, Mendelian randomization; DR, Diabetic retinopathy. Scatter plot of different Mendelian randomization model effect estimates of NOG on diabetic retinopathy.

Owing to the presence of only one SNP in each of the 37 proteins, subsequent sensitivity analysis was not feasible. As a result, we obtained pQTL files for the 37 proteins from the deCODE database for further analysis. According to the cis-pQTL selection criteria for this 37-filtered-proteins pQTL file, get 35 protein to cis - pQTL (see Appendix **Supplementary Table S3**), and then of the 35 through two-sample MR protein and analyses using two-sample MR with DR. For the secondary screening, we used the more strict inspection for with the Bonferroni correction, in which p.adjust < 0.05 was the significant causal filter condition, to determine whether DR had any strong causal associated proteins. The findings presented in **Table 2** indicate a causal relationship between a specific protein and DR, with noggin (NOG) protein showing a negative correlation with the risk of developing the condition. Finally, we created a scatter diagram showing the MR effect of NOG protein and DR (**Figure 2**), showing that each model line on the vertical intercept tends toward zero, and the slopes are all in the same direction.

**Table 2:** Mendelian randomization causal effect estimates of druggable proteins on the onset of DR from deCODE.

exposure	outcome	nsnp	b	se	OR (95% CI)	p.adjust	method
CFH	Diabetic retinopathy	1	0.036337	0.066134	1.04 (0.91, 1.18)	1	Wald ratio
RTN4R	Diabetic retinopathy	4	0.107451	0.045161	1.11 (1.02, 1.22)	0.589775	Inverse variance weighted
CHRDL2	Diabetic retinopathy	4	0.08449	0.077789	0.92 (0.79, 1.07)	1	Inverse variance weighted
LAMC2	Diabetic retinopathy	1	0.036124	0.038645	1.04 (0.96, 1.12)	1	Wald ratio
FGFR3	Diabetic retinopathy	2	0.19247	0.070028	0.82 (0.72, 0.95)	0.203554	Inverse variance weighted
CD59	Diabetic retinopathy	3	0.107684	0.089652	1.11 (0.93, 1.33)	1	Inverse variance weighted
NID2	Diabetic retinopathy	4	0.08841	0.035632	1.09 (1.02, 1.17)	0.445223	Inverse variance weighted
CTSH	Diabetic retinopathy	5	0.04626	0.02296	1.05 (1.00, 1.10)	1	Inverse variance weighted
HSPB1	Diabetic retinopathy	3	0.07702	0.051606	1.08 (0.98, 1.20)	1	Inverse variance weighted
BST1	Diabetic retinopathy	9	0.01613	0.017309	0.98 (0.95, 1.02)	1	Inverse variance weighted
CRTAM	Diabetic retinopathy	5	0.07001	0.073212	0.93 (0.81, 1.08)	1	Inverse variance weighted
ENPP5	Diabetic retinopathy	3	0.01044	0.071669	0.99 (0.86, 1.14)	1	Inverse variance weighted
ADAM23	Diabetic retinopathy	3	0.04646	0.038872	0.95 (0.88, 1.03)	1	Inverse variance weighted
LY9	Diabetic retinopathy	5	0.026926	0.027517	1.03 (0.97, 1.08)	1	Inverse variance weighted
CPA4	Diabetic retinopathy	4	0.0138	0.015279	0.99 (0.96, 1.02)	1	Inverse variance weighted
DUT	Diabetic retinopathy	1	0.115906	0.223181	1.12 (0.73, 1.74)	1	Wald ratio
PPT1	Diabetic retinopathy	2	0.074389	0.130506	1.08 (0.83, 1.39)	1	Inverse variance weighted
CHL1	Diabetic retinopathy	5	0.0815	0.05138	0.92 (0.83, 1.02)	1	Inverse variance weighted
CFHR4	Diabetic retinopathy	8	0.024325	0.023215	1.02 (0.98, 1.07)	1	Inverse variance weighted
CPM	Diabetic retinopathy	2	0.23203	0.112093	0.79 (0.64, 0.99)	1	Inverse variance weighted
COL6A1	Diabetic retinopathy	8	0.00681	0.02485	0.99 (0.95, 1.04)	1	Inverse variance weighted
F13B	Diabetic retinopathy	2	0.02774	0.159561	0.97 (0.71, 1.33)	1	Inverse variance weighted
PAM	Diabetic retinopathy	2	0.04469	0.035197	0.96 (0.89, 1.02)	1	Inverse variance weighted
RNASE3	Diabetic retinopathy	5	0.057573	0.042397	1.06 (0.97, 1.15)	1	Inverse variance weighted
CST5	Diabetic retinopathy	2	0.050544	0.031303	1.05 (0.99, 1.12)	1	Inverse variance weighted
NEGR1	Diabetic retinopathy	1	0.26061	0.259151	0.77 (0.46, 1.28)	1	Wald ratio
WFIKKN2	Diabetic retinopathy	5	0.040372	0.031884	1.04 (0.98, 1.11)	1	Inverse variance weighted
NQO1	Diabetic retinopathy	3	0.059827	0.023121	1.06 (1.01, 1.11)	0.328598	Inverse variance weighted
COL18A1	Diabetic retinopathy	2	0.118148	0.101691	1.13 (0.92, 1.37)	1	Inverse variance weighted
NOG	Diabetic retinopathy	3	0.15746	0.045523	0.85 (0.78, 0.93)	0.018434	Inverse variance weighted
GP1BA	Diabetic retinopathy	3	0.116146	0.08927	1.12 (0.94, 1.34)	1	Inverse variance weighted
PLA2G2A	Diabetic retinopathy	3	0.03449	0.022879	0.97 (0.92, 1.01)	1	Inverse variance weighted
GSTA1	Diabetic retinopathy	5	0.092417	0.039726	1.10 (1.01, 1.19)	0.679957	Inverse variance weighted
CFHR1	Diabetic retinopathy	4	0.032898	0.036886	1.03 (0.96, 1.11)	1	Inverse variance weighted

SNP, single nucleotide polymorphism; OR, odds ratio; CI, confidence interval.

### 2.3 Sensitivity analysis of protein and DR

We conducted a heterogeneity analysis between NOG protein and DR (**Table 3**). The findings indicated that there was no significant heterogeneity in the MR results for NOG protein related to DR (I² = 0, Cochran Q p.adjust > 0.05). Subsequently, we carried out pleiotropy assessments for NOG protein and DR (**Table 4**). The results presented in the table demonstrate that the p.adjust values for pleiotropy tests of all proteins exceeded 0.05, and the intercepts were near zero. This suggests that horizontal pleiotropy did not influence the causal inference.

**Table 3:** Heterogeneity test of Mendelian randomization analysis of proteins on diabetic retinopathy.

exposure	outcome	Q	Q_df	Q_pval	I^2^(%)
NOG	Diabetic retinopathy	1.353852724	2	0.508176545	0

Q, Cochran Q test statistic; Q_df, Q test degree of freedom; Q_pval, Q test P values; I^2^ statistics reflect the heterogeneity of instrumental variable part of the proportion of the total variance in:^2–^0 or less, I set it to 0, showed no observed heterogeneity; I^2^ = 0 - 25%, suggesting mild heterogeneity; I^2^ = 25%-50%, indicating moderate heterogeneity; I^2^>50% indicated high heterogeneity. The specific calculation formula is^2^ I=(q-df)/Q×100%.

**Table 4:** Mendelian randomization analysis level pleiotropy test of proteins for diabetic retinopathy.

exposure	outcome	egger_intercept	se	pval
NOG	Diabetic retinopathy	0.063481801	0.054578184	0.452079369

Sensitivity analysis of the results with the use of one-by-one exclusion tests did not show a significant change in the estimates of the protein NOG effect, suggesting stability of the results (Appendix **Supplementary Table S4**). Leave-one-out analysis was used to remove each instrumental variable and examine the causal effect of NOG protein on DR, and no significant deviation was found from the lump effect of instrumental variables. To ensure that the causal effect of protein on the pathogenesis of DR was in the correct direction, we used Steiger directionality test for analysis. We found that the p.adjust for NOG protein and DR was far less than 0.05, indicating the correct direction (**Table 5**).

**Table 5:** Protein Mendelian randomization analysis of diabetic retinopathy: Steiger directional inspection.

exposure	outcome	snp_r2.exposure	snp_r2.outcome	correct_causal_direction	steiger_pval
NOG	Diabetic retinopathy	0.029828269	6.14 e-05	TRUE	1.42 e-185

### 2.4 SMR analysis and colocalization analysis

We aimed to gather further evidence through the analysis of the SMR pleiotropic presence. **Table 6** shows that the SMR analysis results of NOG protein p_SMR < 0.05, indicating a causal relationship. Based on the results of colocalization analysis (**Table 7**), we observed a relationship between NOG and DR (PP.H4 > 0.8).

**Table 6:** Results of SMR analysis of proteins for diabetic retinopathy.

Gene	exposure	outcome	topSNP	b_SMR	se_SMR	p_SMR
ENSG00000183691.4	NOG	Diabetic retinopathy	rs76164057	0.173837	0.0487455	3.62 e-04

SMR, Summary-data-based Mendelian Randomization. SNP, single nucleotide polymorphism.

**Table 7:** Results of colocalization analysis of protein and diabetic retinopathy.

exposure	outcome	PP.H0.abf	PP.H1.abf	PP.H2.abf	PP.H3.abf	PP.H4.abf
NOG	Diabetic retinopathy	4.9022 e-260	0.08559018	1.437 e-260	0.024	0.890

### 2.5 Drug targets

**Figure 3:**
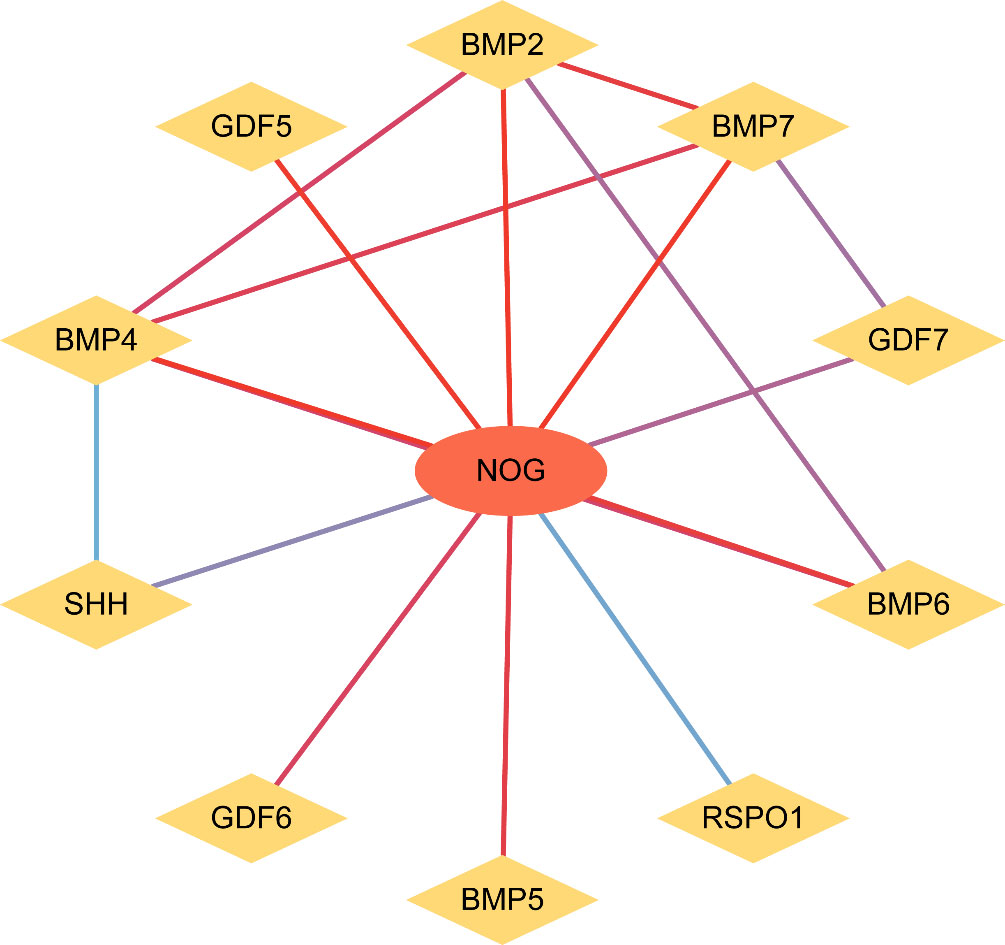
PPI network analysis. PPI network, Protein - Protein interaction network. In the figure, nodes represent proteins, and the color of the line from blue to red indicates the degree of correlation between nodes from small to large.

We extended the PPI analysis of druggable targets (such as NOG) using the STRING database and constructed a network of 10 related proteins (BMP2, BMP4, BMP5, BMP6, BMP7, GDF5, GDF6, GDF7, SHH, SHH, BMP2, BMP4, BMP5, BMP6, and BMP7) after retaining the targets that had connections with other nodes. RSPO1) in the protein-protein interaction network (**Figure 3**) We used 11 target proteins to analyze the potential drugs of target proteins by using the DURGBANK database. **Table 8** shows that BMP4 encoded proteins corresponding to DB01373 drugs, NOG, BMP2, BMP5, BMP6, BMP7, GDF5, GDF6, GDF7, SHH, DB01373 drugs, NOG, BMP2, BMP5, BMP6, BMP7, GDF5, GDF6, GDF7, SHH, there is no corresponding drug for RSPO1 encoded protein, which could be further explored. We then used DSigDB on the Enrichr platform to screen results, with p.adjust <0.05 (**Table 9**), and we identified 00006624 estradiol and progesterone CTD CTD 00005920 NOG as potential drugs.

**Table 8:** Drug information of patent drug-related targets in DRUGBAN.

target	uniprot	drugbank ID	name	durg group	Pharmacological action	actions
BMP2	P12643	NA	NA	NA	NA	NA
BMP4	P12644	DB01373	Calcium	nutraceutical	unknown	NA
BMP5	P22003	NA	NA	NA	NA	NA
BMP6	P22004	NA	NA	NA	NA	NA
BMP7	P18075	NA	NA	NA	NA	NA
GDF5	P43026	NA	NA	NA	NA	NA
GDF6	Q6KF10	NA	NA	NA	NA	NA
GDF7	Q7Z4P5	NA	NA	NA	NA	NA
NOG	Q13253	NA	NA	NA	NA	NA
SHH	Q15465	NA	NA	NA	NA	NA
RSPO1	Q2MKA7	NA	NA	NA	NA	NA

**Table 9:** Predicted drug candidates/compounds using DSigDB.

Term	P.value	Adjusted.P.value	Old.P.value	Old.Adjusted.P.value	Odds.Ratio	Combined.Score	Genes
Octreotide CTD 00007059	4.36 e-10	8.55 e-08	0	0	600.6015	12944.95	BMP4; BMP2; BMP7; BMP6
TITANIUM BOSS	1.89 e-07	1.85 e-05	0	0	118.4107	1833.191	BMP4; BMP2; BMP7; BMP6
Nandrolone phenpropionate BOSS	3.81 e-07	2.49 e-05	0	0	98.7528	1459.7	BMP4; BMP2; BMP7; BMP6
Electrocorundum CTD 00005364	4.38 e-06	0.000214	0	0	128.8642	1590.068	BMP4; BMP2; BMP6
triclosan CTD 00006933	6.36 e-06	0.000249	0	0	113.1989	1354.532	BMP2; GDF5; BMP6
Heparitin BOSS	1.42 e-05	0.000463	0	0	85.78448	957.7644	BMP4; BMP2; BMP7
4-(2-Aminoethyl)benzenesulfonyl fluoride CTD 00000065	2.14 e-05	0.000599	0	0	403.596	4339.904	BMP2; SHH
Deacetylchitin BOSS	5.66 e-05	0.001387	0	0	53.16696	519.9225	BMP2; BMP7; BMP6
Monoisoamyl-2,3-dimercaptosuccinate CTD 00003178	0.000162	0.003175	0	0	134.3838	1172.878	BMP2; BMP5
Stannic fluoride BOSS	0.000162	0.003175	0	0	134.3838	1172.878	BMP2; BMP7
Arsenenous acid CTD 00000922	0.000359	0.006401	0	0	12.2007	96.76978	BMP4; BMP2; SHH; BMP6; BMP5
Chromium(III) oxide CTD 00001091	0.000511	0.008342	0	0	73.81111	559.4599	BMP4; BMP2
Calcium phosphate BOSS	0.000561	0.008348	0	0	70.28571	526.1064	BMP2; BMP7
Chromium(II) chloride CTD 00000877	0.000596	0.008348	0	0	68.11624	505.7478	BMP4; BMP2
ellipticine PC3 UP	0.00087	0.01137	0	0	56.00563	394.663	BMP4; BMP2
Lead(II) acetate CTD 00000394	0.000935	0.011458	0	0	53.94851	376.2673	BMP4; BMP2
dexamethasone CTD 00005779	0.001347	0.015535	0	0	17.55772	116.0492	BMP4; BMP2; BMP6
Ethylene dimethacrylate BOSS	0.001833	0.019964	0	0	38.07088	239.906	BMP4; BMP2
progesterone CTD 00006624	0.002257	0.023282	0	0	7.887871	48.06658	BMP2; SHH; NOG; BMP7; BMP6
estradiol CTD 00005920	0.003211	0.031469	0	0	6.330561	36.34456	BMP4; BMP2; SHH; NOG; BMP7; BMP6; BMP5
liothyronine CTD 00006943	0.003418	0.031904	0	0	27.54028	156.3907	BMP2; BMP6
arsenite CTD 00000779	0.004051	0.036091	0	0	8.242063	45.40385	BMP4; GDF6; GDF5; BMP6
8-Bromo-cAMP, Na CTD 00007044	0.004678	0.039866	0	0	11.17488	59.95155	BMP4; RSPO1; BMP6
Acid red 87 BOSS	0.005057	0.041221	0	0	22.44104	118.6446	BMP2; BMP7
dexamethasone BOSS	0.005258	0.041221	0	0	21.98778	115.3929	BMP4; BMP2
celastrol MCF7 UP	0.006707	0.049927	0	0	19.34606	96.82027	BMP4; BMP7
Retinoic acid BOSS	0.006878	0.049927	0	0	19.09082	95.06204	BMP4; BMP2

### 2.6 Validation of the expression discrepancies of key genes in the normal group and diseases group

**Figure 4:**
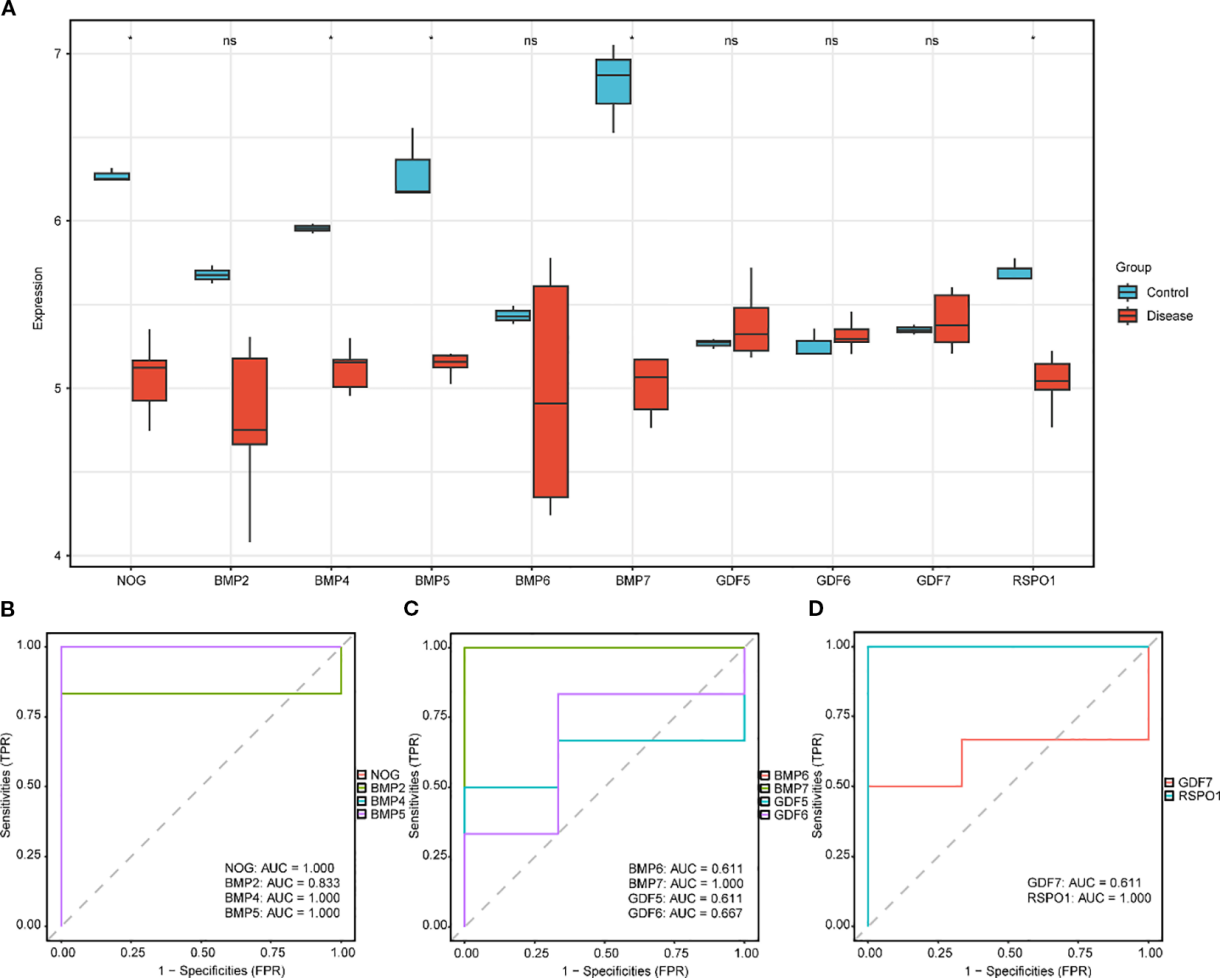
Differential expression validation of key genes in GSE60436 in DR and normal group. **(A)** The group comparison graph of key genes (Key Genes) in the diabetic retinopathy (DR) group and the normal (Normal) group in GSE60436. B-D. The ROC curves of key genes (Key Genes) NOG, BMP2, BMP4 and BMP5 **(B)**, BMP6, BMP7, GDF5 and GDF6 **(C)**, GDF7 and RSPO1 **(D)** in the DR group. DR, diabetic retinopathy; ROC, Receiver Operating Characteristic; AUC, Area Under the Curve; TPR, True Positive Rate; FPR, False Positive Rate. * Denotes p value < 0.05, which is statistically significant; ns denotes p value ≥ 0.05, which is not statistically significant. In the group comparison graph, blue represents the normal (Normal) group and red represents the DR group. When the AUC exceeds 0.5, it suggests that the molecule’s expression is associated with a trend that promotes the event’s occurrence. As the AUC approaches 1, the diagnostic performance improves. An AUC in the range of 0.5 to 0.7 indicates limited accuracy, while an AUC between 0.7 and 0.9 suggests moderate accuracy. An AUC greater than 0.9 signifies high accuracy.

To investigate the expression variations of key genes (Key Genes) in the GEO dataset GSE60436 (**Figure 4**), the differential analysis results of the expression levels of the 10 key genes (Key Genes) in the DR group and the normal (Normal) group in the GEO dataset GSE60436 were presented via group comparison graphs and ROC curves. The differential results revealed that the expression quantities of the 10 key genes (Key Genes) in the DR group and the Normal group of the GEO dataset GSE60436 were dissimilar. In the GEO dataset GSE60436, the expressions of the key genes (Key Genes) NOG, BMP4, BMP5, BMP7, and RSPO1 exhibited significant differences (pvalue < 0.05).

Ultimately, the ROC curves were plotted based on the expression levels of the key genes (Key Genes) in the DR group using the R package pROC. In the GEO dataset GSE60436 (**Figures 1B-D**), the ROC curves demonstrated that the expression levels of the key genes (Key Genes) NOG, BMP4, BMP5, BMP7, and RSPO1 in the DR group presented high accuracy (AUC > 0.9) in discriminating between different groups; the expression level of the key gene (Key Genes) BMP2 in the DR group presented certain accuracy (0.7 < AUC < 0.9) in discriminating between different groups; and the expression levels of the key genes (Key Genes) BMP6, GDF5, GDF6, and GDF7 in the DR group presented low accuracy (0.5 < AUC < 0.7) in discriminating between different groups.

### 2.7 Immune infiltration analysis

**Figure 5:**
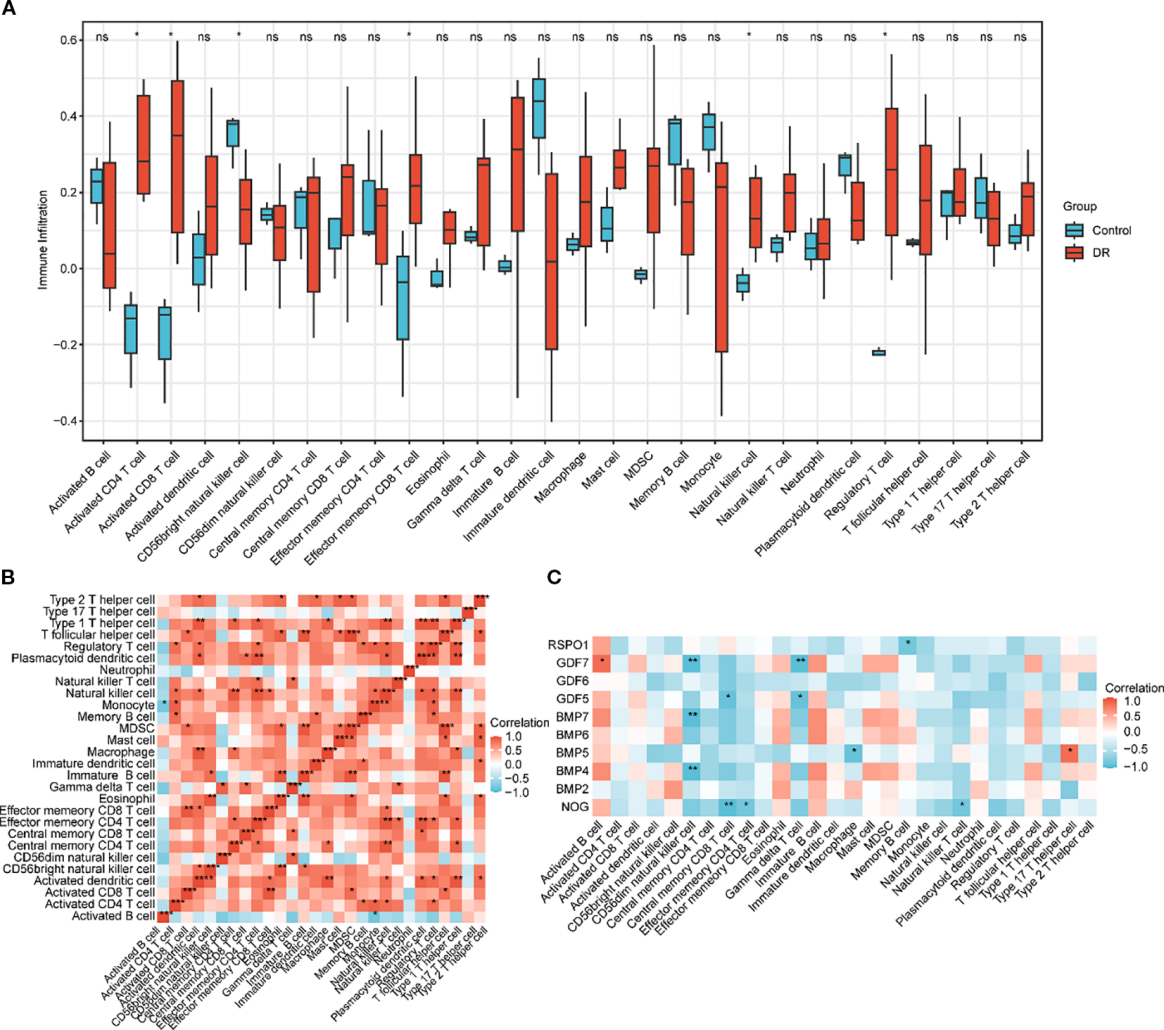
Immune infiltration analysis by ssGSEA algorithm. **(A)** Group comparison chart of immune cells in the DR group and the normal (Normal) group in the GEO dataset GSE60436. **(B)** Correlation heatmap of immune cell infiltration abundance in the GEO dataset GSE60436. **(C)** Correlation heatmap of key genes (Key Genes) with immune cell infiltration abundance in the GEO dataset GSE60436. ssGSEA, single-sample Gene-Set Enrichment Analysis; DR, diabetic retinopathy. ns indicates p value ≥ 0.05, no statistical significance; * indicates p value < 0.05, statistically significant; ** indicates p value < 0.01, highly statistically significant; *** indicates p value < 0.001, extremely statistically significant. A correlation coefficient (r value) absolute value below 0.3 is considered weak or not correlated, between 0.3 and 0.5 is weakly correlated, between 0.5 and 0.8 is moderately correlated, and above 0.8 is strongly correlated. In the group comparison chart, blue represents the normal (Normal) group and red represents the DR group. Red indicates positive correlation and blue indicates negative correlation. The depth of color represents the strength of the correlation.

The expression matrix of the GEO dataset GSE60436 was employed to calculate the immune infiltration abundance of 28 types of immune cells via the ssGSEA algorithm. Firstly, the expression discrepancies of the immune cell infiltration abundance among different groups were displayed through a group comparison chart (**Figure 5A**). The results demonstrated that the infiltration abundances of six immune cells, including Activated CD4+ T cell, Activated CD8+ T cell, CD56bright natural killer cell, Effector memory CD8 +T cell, Natural killer T cell, and Regulatory T cell exhibited statistically significant differences between the DR group and the normal (Normal) group (p value < 0.05). Subsequently, the correlation outcomes of the infiltration abundance of 28 immune cells in the GEO dataset GSE60436 were presented via a correlation heatmap (**Figure 5B**). The results revealed that the majority of immune cells were positively correlated. Then, the correlations between 10 key genes and 28 immune cells were analyzed and presented through a correlation heatmap (**Figure 5C**). The results indicated that there was the strongest positive correlation between the key gene GDF6 and the immune cell Activated B cell (r value = 0.82, p value < 0.05), and between the key gene BMP5 and the immune cell Type 17 T helper cell (r value = 0.82, p value < 0.05); the strongest negative correlation was observed between the key gene GDF7 and the immune cell CD56dim natural killer cell (r value = -0.94, p value < 0.01), between the key gene BMP7 and the immune cell CD56dim natural killer cell (r value = -0.94, p value < 0.01), between the key gene BMP4 and the immune cell CD56dim natural killer cell (r value = -0.94, p value < 0.01), between the key gene NOG and the immune cell Central memory CD8 T cell (r value = -0.94, p value < 0.01), and between the key gene GDF7 and the immune cell Gamma delta T cell (r value = -0.94, p value < 0.01).

### 2.8 Laboratory verification

**Figure 6:**
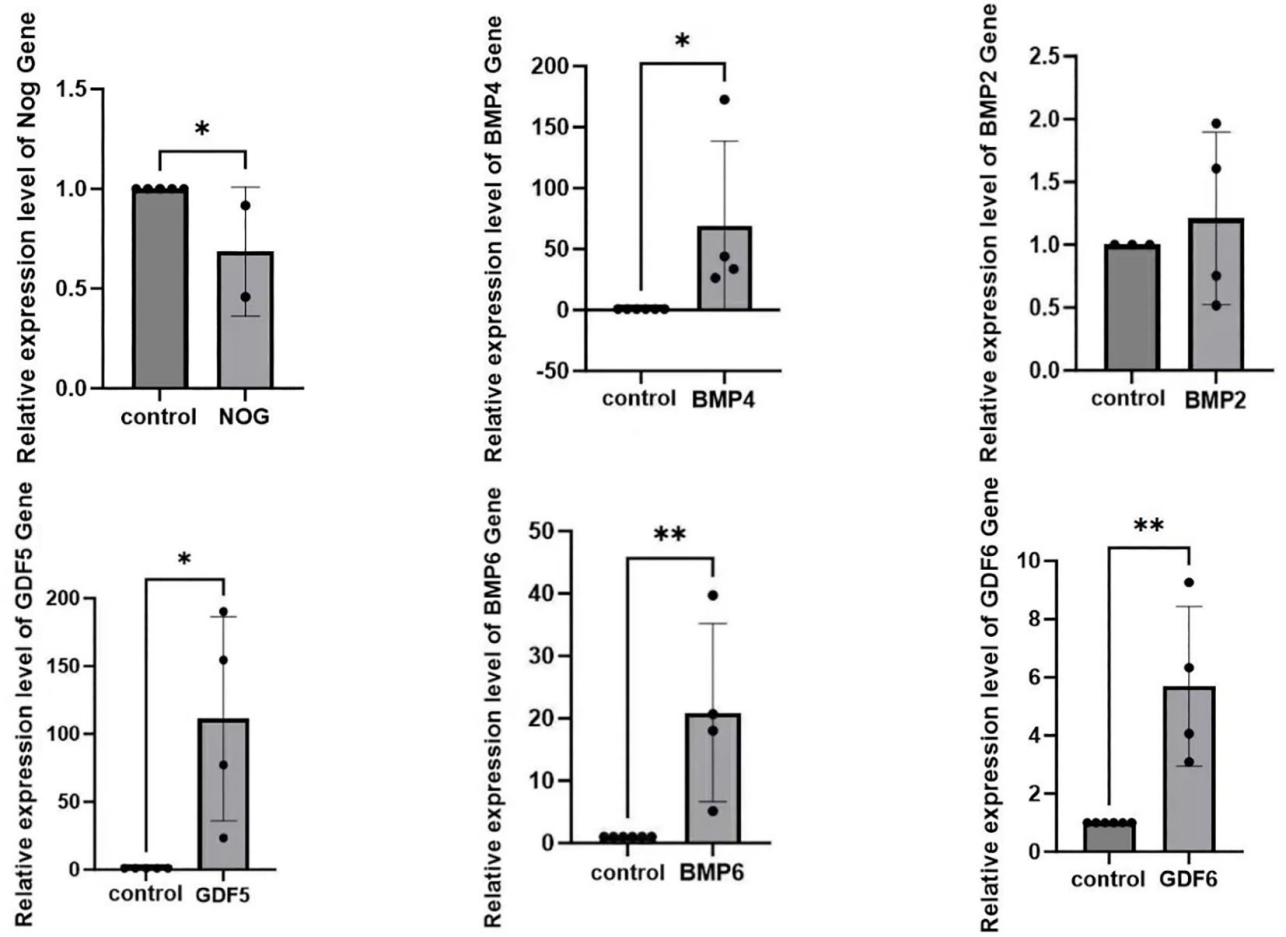
The discrepancy of NOG, BMP4, BMP2, GDF5, BMP6, GDF6 expression in the blood between patients with diabetic retinopathy and normal individuals(*p<0.05).

We extracted blood from six patients with DR and six healthy individuals. RNA was isolated from the blood and reverse transcribed into cDNA, which was subsequently amplified using a PCR apparatus. The obtained results were subjected to statistical analysis via SPSS 18.0 (SPSS, Chicago, IL), revealing that the expression of NOG protein in the blood of patients with DR was significantly decreased compared to the normal group and the expression levels of BMP2, BMP4, BMP6, GDF5, and GDF 6 were significantly elevated (**Figure 6**).

### 2.9 Molecular docking

**Figure 7:**
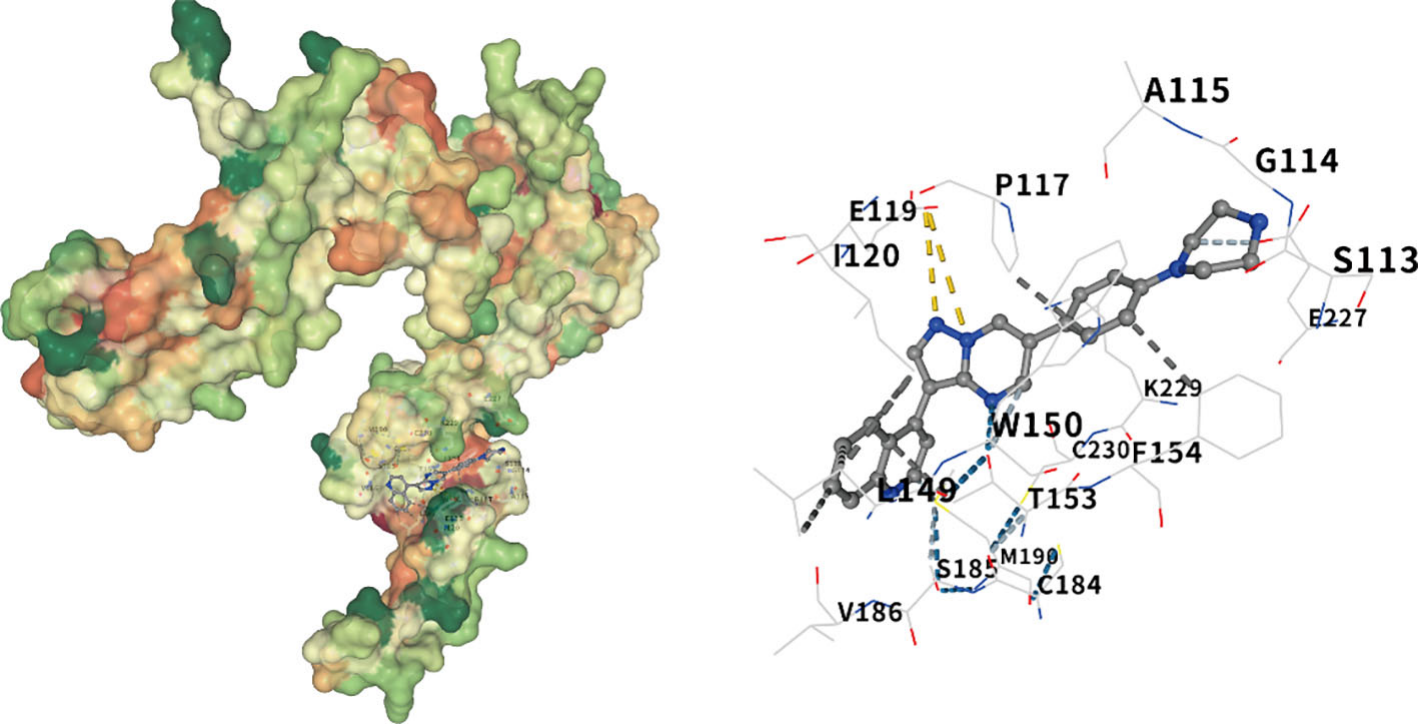
Molecular docking of noggin and LDN-193189. The visualization of the docking results of Noggin protein and LDN-193189, from left to right, are the global docking map and the interaction force map. The color of the protein surface gradually changes from green, orange to red, indicating the change of amino acid properties from hydrophilic to hydrophobic. Blue dotted line — hydrogen bond, light blue dotted line — weak hydrogen bond, gray dotted line — hydrophobic interaction force, cyan dotted line — halogen bond, yellow dotted line — ionic bond.

Molecular docking of the Noggin protein encoded by the NOG gene and the BMP pathway inhibitor LDN-193189 was performed using CB-Dock2. The docking results between the Noggin protein and its corresponding active component are presented in **Figure 4**. The interaction analysis revealed a strong binding affinity between Noggin and LDN-193189, with a Vina Score of -9.4 Kcal/mol. Specifically, amino acids SER113, GLY114, ALA115, MET116, PRO117, SER118, GLU119, ILE120, LEU123, LEU149, TRP150, THR153, PHE154, CYS155, VAL157, TYR159, CYS184, SER185, VAL186, PRO187, MET190, SER195, LYS196, SER226, GLU227, CYS228, LYS229, and CYS230 participate in the molecular interactions through hydrogen bonds, ionic bonds, and hydrophobic interactions (**Figure 7**).

## 3 Discussion

DR is a serious complication of diabetes that profoundly impacts patients’ quality of life and imposes a substantial social and economic burden. To address the limitations of current treatment options, we aimed to identify new drug targets for DR. The strength of our study lies in the utilization of two-sample MR analysis, which enables a more precise evaluation of the causal relationship between proteins and the disease. On the basis of the aforementioned work, we incorporated external dataset validation, immune infiltration analysis, and laboratory experiments to verify the correlation between the NOG protein and DR.

In this study, we leveraged a multi-faceted bioinformatics approach. Key findings include the selection of genes with therapeutic potential from a pool of 4479 druggable genes, the identification of proteins related to DR through pQTL data analysis, and the determination of causal relationships between several proteins and the disease using two-sample Mendelian randomization (**Tables 1**, **2**). We conducted a sensitivity analysis to establish the causal relationship between the proteins and DR (**Tables 3**–**5**). Further validation was provided by performing SMR and colocalization analyses (**Tables 6**, **7**). The construction of a PPI network and subsequent drug target analysis using DRUGBANK and DSigDB databases have highlighted potential targets for future treatment strategies (**Tables 8**, **9**). These systematic explorations offer a scientific basis for developing new therapeutic approaches for DR. Our results indicate a robust inverse association between NOG protein and DR, and we rigorously validated these results through diverse sensitivity analyses.

Our study’s identification of NOG protein as a negative regulator of DR is supported by previous research indicating its involvement in angiogenesis and fibrosis, processes central to DR pathology. Several researchers have reported that NOG protein represents a potential protective factor against diabetes complications, including retinopathy (10, 11), which aligns with our findings (b=-0.15746, OR = 0.85, p.adjust=0.018434). These studies, which used animal models and patient-derived samples, reported that modulation of NOG expression influences vascular and fibrotic pathways (12, 13). Our team, through the validation of external datasets, immune infiltration analysis and experiments, has demonstrated a negative correlation between the NOG protein and DR. Conversely, some earlier studies reported no association between NOG protein levels and diabetic complications (14), which might be attributed to differences in study design, sample size, or population genetics.

The NOG protein is a dimeric glycoprotein that is secreted and encoded by the NOG gene, with a molecular weight of 64 kDa. The NOG protein is recognized for its role in regulating BMPs (15). Darwish et al. demonstrated an upregulation of BMP4 expression in diabetic human patients as well as mice, which was found to impair the function of the human retinal endothelial barrier. Furthermore, NOG acts as an inhibitor of BMP/ALK signal transduction to mitigate the detrimental effects of BMP4 (12). Using Akita mice as a model, Humble J et al. conducted immunofluorescence analysis of BMP4 and lectins in the eyes of humans and mice with and without diabetes. The expression of BMP4 was found to be three times higher in samples from patients with diabetes, and NOG attenuated the effects of BMP4 on retinal endothelial cells (16). The upregulation of BMP2 has been demonstrated in the retinas of experimental diabetic animal models and human patients with diabetes. It has also been shown that BMP2 is inhibited by NOG. The inhibition of BMP2 signaling has been found to preserve retinal endothelial cell barrier function in individuals with hyperglycemia (17). The literature collectively suggests that NOG, as a BMP inhibitor, effectively preserves the functional integrity of retinal endothelial barrier and reduces the occurrence of DR. This indirectly aligns with our experimental findings. Nevertheless, no direct studies have reported the association between NOG and DR prior to our investigation.

In conclusion, BMP is increasingly acknowledged as a multifunctional regulator of angiogenesis, tissue homeostasis, and tumorigenesis. The activation of BMP signal transduction activity occurs in various disease contexts. There are four BMP-1 receptors: Alk1/Acvrl1, Alk2/Acvr1, Alk3/Bmpr1a, and Alk6/Bmpr1b. BMP9/10, which exhibits anti-angiogenic effects, demonstrates a higher affinity for ALK1. The absence of ALK1 results in vigorous angiogenesis, indicating that ALK1 regulates the vascular inhibitory effect of BMP9/10 in endothelial cells. Furthermore, ALK2, ALK3, and ALK6 bind to BMP2, BMP4, and BMP6, respectively, to regulate angiogenic signaling. Additionally, NOG protein serves as an inhibitory regulator of BMP and could effectively inhibit the occurrence of DR. It could also serve as an early diagnostic criterion for DR.

Our MR analysis has provided robust evidence for the involvement of NOG protein in DR, suggesting a protective effect against this complication of diabetes. Consistent estimates across multiple models and a lack of significant bias in both heterogeneity and pleiotropy tests reinforce the credibility of this inverse causal relationship. The observed odds ratio of 0.85, with a 95% confidence interval from 0.78 to 0.93 and an adjusted p-value of 0.018434, suggests that elevated levels of NOG protein are linked to a lower risk of developing DR. Mendelian randomization employs genetic variants as instrumental variables to investigate whether risk factors have a causal effect on health outcomes. However, the identified causal relationship could potentially be influenced by reverse causality, horizontal pleiotropy, or genetic confounding due to linkage disequilibrium (LD). To address these concerns, bidirectional MR was performed. The proteins initially identified through MR analysis did not show evidence of reverse causality, a conclusion reinforced by Steiger filtering.This approach ensures that the observed associations are less likely to be confounded by reverse causation or other biases, thereby strengthening the validity of the causal inference.

Further substantiating our findings, summary data-based Mendelian randomization (SMR) analysis yielded a significant p-value (p_SMR = 3.62 e-04), reinforcing the notion that NOG expression exerts a causal influence on the pathogenesis of DR. This is particularly noteworthy as it aligns with previous biological insights into the role of NOG in tissue development and repair processes, which could be pertinent to the vascular and neural components affected in DR.

Colocalization analysis added another layer of support by demonstrating shared genetic etiology between NOG expression and DR (PP.H4.abf = 0.890). This high posterior probability suggests that variants influencing NOG expression are likely to be the same variants associated with disease risk, providing further credence to our hypothesis.

The construction of a PPI network using the STRING database identified several proteins related to bone morphogenetic proteins (BMPs), including BMP2, BMP4, BMP5, BMP6, BMP7; growth differentiation factors (GDFs) like GDF5, GDF6, GDF7; SHH; and RSPO1. These proteins are interconnected and have known roles in angiogenesis and tissue remodeling, processes which are critical in the pathophysiology of DR. In addition, BMPs enhance angiogenesis by promoting endothelial cell migration, invasion, and proliferation. The interaction between BMP-SMAD and Notch signaling pathways plays a critical role in determining endothelial cell stemness during retinal angiogenesis in both embryonic and postnatal development. Furthermore, *in vitro* and *in vivo* studies have shown that BMP2 and BMP4 exert pro-angiogenic effects via the VEGF-A/VEGFR2 and angiopoietin-1/TIE2 signaling pathways. BMP signal transduction also plays a vital role in regulating both physiological and pathological processes in endothelial cells. It has been implicated in various conditions characterized by vascular hyperpermeability, such as acute inflammation and atherosclerosis.Tumor necrosis factor (TNF)-α induces BMP2 expression in human umbilical vein endothelial cells (HUVECs) and chondrocytes through the NFκB pathway, indicating that BMP2 may have pro-inflammatory properties. Elevated BMP2 expression is commonly observed in monocytes from individuals with type 2 diabetes. Moreover, high glucose exposure promotes human macrophages to adopt an M1 inflammatory phenotype. Consequently, increased BMP2 levels in individuals with type 2 diabetes may contribute to heightened inflammatory responses.These findings highlight the multifaceted role of BMPs in both angiogenesis and inflammation, underscoring their significance in various pathological conditions. Our research leverages comprehensive datasets from the deCODE and MRC IEU OpenGWAS databases to enhance the reliability of our findings. Our multifaceted approach combining pQTL data set analysis with GWAS data provides an integrated view of the genetic landscape influencing DR. The use of advanced bioinformatics tools such as the Two Sample MR package for Mendelian randomization analysis further substantiates the credibility of our results.

After identifying the above-mentioned proteins, we conducted a drug/compound - gene association analysis and determined that progesterone CTD 00006624 and estradiol CTD 00005920 are potential drugs for NOG. In our clinical work, we often find that DR in men usually occurs earlier, is more severe, and has a worse prognosis than in women. We consider this might be related to the levels of estradiol and progesterone. Chen Ying et al. conducted research on female and male mice and found that diabetes inflicts less neurovascular damage in females. They also performed experiments on human females and observed that the prevalence of DR in premenopausal women is significantly reduced. This study strongly emphasizes the importance of estradiol in protecting the retina (18). Regarding this aspect, our team will further conduct clinical research.

In order to gain deeper insights into the relationship between NOG protein and DR, our research group isolated RNA from blood samples obtained from patients with confirmed diagnoses of DR. Following this, we carried out reverse transcription and subsequently performed quantitative PCR analysis on the extracted RNA. The results indicated a significant reduction in the expression of NOG protein among patients with DR, while the expression levels of BMP2, BMP4, BMP6, GDF5, and GDF6 were notably elevated. Although the sample size was limited, our observations aligned well with those derived from earlier bioinformatics studies, thereby strengthening the link between NOG protein and the development of DR. Additionally, our findings validated previously reported experimental data, establishing a negative correlation between NOG protein expression and the expression levels of BMP2, BMP4, BMP6, GDF5, and GDF6. Collectively, these outcomes provide support for the notion that NOG protein could act as a protective factor in the context of DR. Of course, we cannot directly determine the regulation of NOG on the retina by collecting NOG mRNA from blood. However, blood sampling is a relatively acceptable method for patients. Next, we may collect vitreous humor for further experiments to increase the credibility of the experiment.

To obtain more potential drugs that may act on the NOG protein, we conducted molecular docking of the NOG protein with BMP inhibitors and found that LDN-193189 has a strong binding force with the NOG protein. This indicates that the NOG protein and LDN-193189 have strong structural complementarity. Their strong binding may alter the function and role of the NOG protein, which might provide some new ideas for the treatment of DR.

Despite the promising results obtained, this study has several limitations that should be acknowledged. First, the laboratory experiments conducted had a limited number of samples, which may introduce potential biases. These experiments are essential for verifying computational predictions in a biological setting. Second, the relatively small sample size could affect the reliability and broader applicability of the findings. Third, the absence of clinical validation represents a significant gap, as such validation is critical for corroborating the study’s outcomes. More importantly, this study primarily utilized the GSE60436 dataset to analyze the correlation between key genes and immune cells. However, the lack of validation across additional datasets constitutes a limitation of the current analysis.

In conclusion, we successfully identified potential drug targets and associated drugs for the treatment of DR through a comprehensive bioinformatics approach. By integrating pQTL data, GWAS data, and PPI networks, we pinpointed proteins with causal relationships to DR and high colocalization probabilities. The identified drug candidates provide a promising starting point for future therapeutic development. These findings could pave the way for more targeted and effective treatments for DR, pending further validation through wet lab experiments and clinical trials.

## 4 Methods

### 4.1 Druggable gene selection

We learned from the druggable genome and support for target identification and validation in drug development (19) for medicine, a total of 4479 genes (Appendix **Supplementary Table S1**). We then divided the genes into three groups based on their properties and functions. The first group comprised 1427 genes, which included the efficacy targets of approved small molecule and biological therapeutic drugs, as well as those of drug candidates in clinical stages. The second group consisted of 682 genes that encoded targets with known bioactive small molecule binding partners and exhibited at least 50% sequence identity to approved drug targets. The third group included 2370 genes encoding proteins or extracellular proteins that showed lower similarity to approved drug targets and were not part of the first or second groups. These genes represent potential drug targets that have not yet been extensively explored.This classification helps to stratify genes based on their relevance and potential for therapeutic intervention, highlighting those with established therapeutic significance and those that warrant further investigation.

### 4.2 pQTL dataset

To investigate the correlation between genetic mutations and gene expression using protein expression as a trait, we employed protein quantitative trait loci (pQTL) analysis. For our preliminary analysis, we utilized cis-pQTL data reported by Zheng et al. (20), which included 738 cis-SNPs associated with 734 proteins (see Appendix **Supplementary Table S2**). This dataset helped us screen for pharmaceutically viable proteins for further investigation. Subsequently, we accessed filtered protein pQTL data from a large-scale integration of the plasma proteome with genetics and diseases (20), specifically using the deCODE 4674 protein database. These data served as the primary pQTL resource to identify potential drug targets for DR.The selection criteria for cis-pQTL variants were as follows: adjusted p-value < 5e-08; exclusion of SNPs within the major histocompatibility complex (MHC) region; and identification of SNPs located within 500KB upstream or downstream of the gene, while removing those with linkage disequilibrium r² < 0.001. The selected datasets were derived from individuals of European ancestry.This approach allowed us to systematically evaluate genetic associations with protein expression, thereby enhancing our ability to pinpoint promising drug targets for DR.

### 4.3 Outcome dataset

We obtained the GWAS ID of DR (finn-b-DM_RETINOPATHY) from the MRC IEU OpenGWAS (21) database, and standardized association summary statistics were obtained from the R-packet TwoSampleMR (7) for use as outcomes. A total of 14584 DR experimental samples and 20–082 control samples were included.

### 4.4 Two-sample MR

We performed a two-sampleMR analysis using the TwoSampleMR package, with the pharmaceutically available protein studied by Zheng et al (20) as the exposure factor and DR as the outcome. We used Wald thewire method evaluation contains only one SNP exposed Mendelian randomization results, using inverse variance weighted (IVW) method to evaluate the samples containing two or more exposed MR results of SNP. We used TwoSampleMR heterogeneity inspection, pleiotropic test, and a method of analysis, and then used the inspection for a Steiger directionality test to judge the correctness of the causal direction.

After selecting the proteins with significant causal relationships with DR, the pQTL data of the corresponding proteins were downloaded from the deCODE database as the exposure factors and DR as the outcome, and then two-sample MR analysis was performed. Using the same method, we used the Wald thewire method evaluation contains only one SNP exposed Mendelian randomization results, using the IVW method to evaluate contain two or more exposed Mendelian randomization result of SNP. We used TwoSampleMR heterogeneity inspection, pleiotropic test and a method of analysis, and then used the inspection for directional steiger, direction, judged the correctness of the causal direction.

### 4.5 Analysis of SMR

In prior studies, SMR (22) leverages GWAS summary data and expression QTL studies to evaluate pleiotropic associations between baseline protein expression levels and complex traits of interest. The HEIDI (Heterogeneity in Dependent Instruments) test is employed to assess potential horizontal pleiotropy by examining whether there is heterogeneity in the instrumental variable signals. For our analysis, we downloaded the Linux version (1.3.1) of SMR from the official website (https://yanglab.westlake.edu.cn/software/smr) and performed the SMR analysis using default parameters.This approach allows for a robust evaluation of genetic associations between protein expression and complex traits while accounting for potential pleiotropic effects.

### 4.6 Positioning analysis

We utilized the coloc package for conducting colocalization analysis. This package employs a Bayesian approach to evaluate support for five mutually exclusive hypotheses: first, SNP is uncorrelated with trait1 and trait2; second, a relationship exists between SNP and trait1; third, SNP is associated with trait2; fourth, SNP is related to both trait1 and trait2 as independent SNPs; fifth, common SNPs are linked to both trait1 and trait2. The posterior probabilities for each hypothesis test are denoted as H0, H1, H2, H3, and H4, respectively. Each tests the a posteriori probability of H0, H1, H2, H3, and H4. To estimate the shared variable posterior probability, which is chosen for each protein, we retrieved its topSNP upstream and downstream all SNPs within 500 KB for positioning analysis, and we found that the PH4 > 0.8 for GWAS and pQTL provided evidence of positioning.

### 4.7 Drug targets

A protein-protein interaction (PPI) network comprises individual proteins that interact with one another. The STRING database (23) provides a platform for exploring both known and predicted protein interactions. In this study, we utilized the STRING database to construct a PPI network specific to human proteins. We identified proteins that interacted with druggable targets, using a minimum correlation coefficient of greater than 0.900 as the threshold. To build and visualize this network, we employed the R packages `igraph` and `ggraph`.This methodology allowed us to systematically identify and map high-confidence interactions between proteins and druggable targets. By visualizing these interactions, we aimed to gain deeper insights into the functional relationships within the network, facilitating a more comprehensive understanding of potential therapeutic targets.

After that, we searched DRUGBANK (24) and obtained the drugs corresponding to all the proteins contained in PPI and their modes of action. The selected drugs can be used for the later treatment of DR. Characteristics of drug database (DSigDB) is an Enrichr platform (https://maayanlab.cloud/Enrichr/) in the database; it is mainly used for the correlation analysis of drugs and compound with gene expression (25). Understanding the effects of drugs on the expression of specific genes is promising for determining the potential therapeutic effects of existing drugs and compounds in new disease areas, thereby facilitating the discovery and application of new drugs. All the proteins included in PPI were input into DSigDB of the Enrichr platform, and their associations with different drugs and compounds was analyzed.

### 4.8 Statistical methods

All data processing and statistical analyses in this study were performed in the R software environment (version 4.2.2; https://www.r-project.org/). The main analytical methods included two-sample Mendelian randomization (MR), summary-data-based Mendelian randomization (SMR), colocalization analysis (coloc), and protein-protein interaction (PPI) network construction and enrichment analysis. Specifically, in the MR analysis, we used the Wald ratio method (for single instrumental variable) and the inverse variance weighted (IVW) method (for multiple instrumental variables) to estimate the causal relationship between exposure (protein) and outcome (disease). Heterogeneity among instrumental variables was assessed using Cochran’s Q test, while horizontal pleiotropy was evaluated using MR-Egger regression. Sensitivity analysis was conducted via leave-one-out validation. The Steiger test was applied to verify the direction of causality. SMR analysis incorporated the HEIDI test to exclude potential pleiotropic effects and further validate the association between protein and disease. Colocalization analysis was performed using the coloc package with a Bayesian framework to determine whether pQTL and GWAS signals shared the same causal variant (26). The PPI network was constructed using the STRING database with an interaction confidence threshold of > 0.900, and visualized using the igraph and ggraph packages. Drug-gene association analysis was performed using the DSigDB database, with a significance threshold of adjusted p-value (FDR) < 0.05. Unless otherwise stated, the statistical significance level was set at P < 0.05, and multiple testing correction was performed using the Bonferroni method.

### 4.9 The verification of the expression differences of key genes in the normal group and diseases

To further verify the expression differences of key genes in the DR group and the normal group in the GEO dataset GSE60436, group comparison plots were drawn based on the expression levels of key genes. Finally, the R package pROC (27) (Version 1.18.5) was used to draw the ROC curves of key genes and calculate the area under the curve (AUC) values to evaluate the diagnostic efficacy of the expression levels of key genes for the occurrence of DR. The AUC of the ROC curve is generally between 0.5 and 1. The closer the AUC is to 1, the better the diagnostic effect. When the AUC is between 0.5 and 0.7, the accuracy is low; when it is between 0.7 and 0.9, the accuracy is moderate; and when it is above 0.9, the accuracy is high.

### 4.10 Immune infiltration analysis

Single-Sample Gene Set Enrichment Analysis (ssGSEA) (28) is a method used to quantify the relative abundance of immune cell infiltration in individual samples. In this study, we first identified and labeled various human immune cell subtypes, including activated CD8 T cells, activated dendritic cells, γδ T cells, natural killer cells, regulatory T cells, and others. Using ssGSEA, we calculated enrichment scores to represent the relative abundance of each immune cell type in each sample, generating an immune cell infiltration matrix.Next, we utilized the R package `ggplot2` (version 3.4.4) to create comparison plots that highlighted the differences in immune cell expression between the DR group and the normal control group within the GEO dataset GSE60436. Immune cells showing significant differences between these two groups were selected for further analysis.To explore the relationships among immune cells, we computed their correlations using the Spearman algorithm and visualized the results with a heatmap generated by the R package `pheatmap` (version 1.0.12). This heatmap displayed the correlation analysis outcomes among the immune cells themselves.Additionally, we assessed the correlation between model genes and immune cells using the Spearman algorithm, retaining only those results with a p-value < 0.05. Finally, we used `ggplot2` (version 3.4.4) to generate a correlation bubble chart, illustrating the relationships between model genes and immune cells.This comprehensive approach allowed us to systematically analyze and visualize the interactions between immune cells and model genes, providing valuable insights into the immune landscape in DR.

### 4.11 Molecular docking

To further analyze the interaction mechanism between the NOG gene-encoded Noggin protein and the BMP pathway inhibitor: LDN-193189, we conducted molecular docking of the NOG gene-encoded Noggin protein and its corresponding small molecule compound using the CB-Dock2 website. CB-Dock2 is an improved version of the CB-Dock server for protein-ligand blind docking, integrating cavity detection, docking, and homology template fitting. Based on the three-dimensional (3D) structures of the protein and ligand, we predicted their binding sites and affinities, thereby achieving computer-aided drug discovery.

Firstly, we downloaded the molecular structure of the drug LDN-193189 (CID: 25195294) from the PubChem database (https://pubchem.ncbi.nlm.nih.gov). Subsequently, we obtained the X-ray crystal structure of Noggin (PDB code: 1M4U) from the PDB (Protein Data Bank) structure database (https://www.rcsb.org/). Finally, we used the AutoDock vina program on the CB-Dock2 website to perform blind docking and visualization of the NOG gene-encoded Noggin protein and its corresponding small molecule compound. The docking score of AutoDock Vina, Vina Score, indicates the strength of the binding force. A Vina Score > -4 Kcal/mol is considered to have a very weak binding force or no binding force; -7 Kcal/mol < Vina Score < -4 Kcal/mol is defined as a moderate binding force; and a Vina Score < -7 Kcal/mol is defined as a standard with a strong binding force.

### 4.12 Laboratory validation

Patients hospitalized in our hospital in December 2024 were recruited. The research subjects comprised 6 cases in the type 2 DR (DM) group; 6 patients who underwent cataract surgery in our hospital during the same period were selected as the blank control group. 1. Inclusion criteria: All enrolled patients were diagnosed with type 2 DR (DM) after mydriasis and ophthalmoscopy by professional ophthalmologists in our hospital. 2. Exclusion criteria: (1) Type 1 diabetes, special type diabetes or accompanied by acute complications of diabetes; (2) Acute or chronic infections, patients with severe traumas; (3) Tumors, hematological diseases, cardiovascular and cerebrovascular disorders; (4) History of treatment with corticosteroids or immunosuppressants and rheumatism and immune-related diseases or other endocrine and metabolic disorders; (5) Pregnancy, lactation or long-term use of contraceptives; (6) Uveitis, glaucoma, retinal diseases and macular degeneration and other ocular diseases.

Sample pretreatment for RNA extraction from blood samples: The blood samples placed in the collection tubes containing anticoagulants were stored in an -80°C freezer. Thus, the blood samples should be retrieved in advance and thawed at room temperature.

1. Sample processing: Prepare a 1.5 mL centrifuge tube (EP), add 300 μL of whole blood and 800 μL of RNA extraction reagent (Trizol) (Cayin Innovation Biotechnology Co., Ltd.) into it. Vigorously vortex for more than 30 seconds and let it stand on ice at 0°C for 5 minutes.
2. Separation: Add 160 μL of chloroform substitute BCP (Cayin Innovation Biotechnology Co., Ltd.), vigorously vortex for 15 seconds, and let it stand on ice at 0°C for 2 minutes. Then, centrifuge at 12,000 g and 4°C for 10 minutes.
3. Precipitation: Transfer the upper aqueous phase to a new EP tube. Precipitate the RNA in the aqueous phase with isopropanol by adding 400 μL of isopropanol. Let it stand at room temperature for 10 minutes, and centrifuge at 12,000 g and 4°C for 10 minutes. Discard the supernatant carefully while retaining the precipitate on the sides and bottom of the tube.
4. Washing: Wash the RNA precipitate with 75% ethanol (prepared with DEPC water). For every 1 mL of RNAkeyTM Reagent used, add 1 mL of 75% ethanol. Gently mix by pipetting, centrifuge at 12,000 g and 4°C for 10 minutes. Discard the supernatant carefully while retaining the precipitate on the sides and bottom of the tube. Open the tube cap and let it stand at room temperature for 5 to 10 minutes to air-dry the RNA precipitate.
5. Dissolution: Add an appropriate amount of RNase-free H2O or DEPC H2O and gently pipette several times to dissolve the RNA. Store at -80°C.
6. RNA reverse transcription reaction: Utilize the kit (Wuhan Saiwei Biotechnology Co., Ltd.). According to the manual, the preparation of the reverse transcription reaction system must be conducted on ice. Prepare a 20 μL system as per the instructions. Mix well by repeated pipetting. Then, place the samples in the reverse transcription instrument for the reverse transcription reaction (reaction conditions: 5 minutes at 25°C, 20 minutes at 42°C, and 5 seconds at 85°C). The resulting samples are cDNA.
7. Real-time fluorescence quantitative PCR: Employ the kit (Cayin Innovation Biotechnology Co., Ltd.). Based on the manual, prepare the PCR reaction system on ice (see **Table 10** below), mix uniformly, and then promptly transfer it to the PCR instrument. The amplification reaction conditions are carried out as per the instructions.HPRT1 was utilized as the internal reference gene. With β-actin serving as the internal reference gene, the required primer sequences are as follows: β-actin: upstream 5´-AAGGCCAACCGCGAGAA-3´, downstream 5´-ATGGGGGAGGGCATACC-3´; NOG: upstream 5´-CGCCCTGGAGTAATTTCGGA-3´, downstream 5´-GCGGAAGAAAGGCACACAAG-3´; Gdf5: upstream 5´-GCTGGGAGGTGTTCGACATC-3´, downstream 5´-CACGGTCTTATCGTCCTGGC-3´; Gdf6: upstream 5´-CACGAGTACATGCTGTCAATCT-3´, downstream 5´-CGTATTAGCCGACTTGGAAGAC-3´; Bmp2: upstream 5´-ACCCGCTGTCTTCTAGCGT-3´, downstream 5´-TTTCAGGCCGAACATGCTGAG-3´; Bmp4: upstream 5´-ATGATTCCTGGTAACCGAATGC-3´, downstream 5´-CCCCGTCTCAGGTATCAAACT-3´; Bmp6: upstream 5´-AGCGACACCACAAAGAGTTCA-3´, downstream 5´-GCTGATGCTCCTGTAAGACTTGA-3´. The relative expression level of NOG, BMP2,BMP4,BMP6, GDF5, and GDF 6 were calculated by the 2-ΔΔCt method. Three replicate wells were set for each sample, and the average of the three measurement results was taken.
8. Statistical analysis was conducted using SPSS 18.0 (SPSS, Chicago, IL) statistical software. The qPCR measurement results of each group were represented as $\bar{\text{x}}$ ± s. The t-test was employed for the comparison of means between groups. The significance level for statistical tests was set at 0.05. GraphPad Prism 10.1.2 software was utilized for plotting.

**Table 10:** The PCR reaction system.

reagent	volume	final concentration
2×SYBRGreenqPCRMasterMixII	10UL	1×
ForwardPrimer(10uM)	0.4UL	0.20uM
ReversePrimer(10uM)	0.4UL	0.20uM
TemplateDNA	Variable	Asrequired
Nuclease-FreeWater	Upto20uL	

## Data Availability

The original contributions presented in the study are included in the article/**Supplementary Material**. Further inquiries can be directed to the corresponding author.

## References

[1] Yau JWY Rogers SL Kawasaki R Lamoureux EL Kowalski JW Bek T. Global prevalence and major risk factors of diabetic retinopathy. Diabetes Care. (2012) 35:556–64. doi: 10.2337/dc11-1909, PMID: 22301125 PMC3322721

[2] Cheung N Mitchell P Wong TY. Diabetic retinopathy. Lancet. (2010) 376:124–36. doi: 10.1016/S0140-6736(09)62124-3, PMID: 20580421

[3] Vujosevic S Aldington SJ Silva P Hernández C Scanlon P Peto T. Screening for diabetic retinopathy: new perspectives and challenges. Lancet Diabetes Endocrinol. (2020) 8:337–47. doi: 10.1016/S2213-8587(19)30411-5, PMID: 32113513

[4] Martínez-García I Cavero-Redondo I Álvarez-Bueno C Pascual-Morena C Gómez-Guijarro MD Saz-Lara A. Non-invasive skin autofluorescence as a screening method for diabetic retinopathy. Diabetes Metab Res Rev. (2024) 40:e3721. doi: 10.1002/dmrr.3721, PMID: 37672325

[5] Bressler NM Beaulieu WT Maguire MG Glassman AR Blinder KJ Bressler SB. Early response to anti-vascular endothelial growth factor and two-year outcomes among eyes with diabetic macular edema in protocol T. Am J Ophthalmol. (2018) 195:93–100. doi: 10.1016/j.ajo.2018.07.030, PMID: 30077569 PMC6648655

[6] Song SJ Han K Choi KS Ko SH Rhee EJ Park C. Trends in diabetic retinopathy and related medical practices among type 2 diabetes patients: Results from the National Insurance Service Survey 2006–2013. J Diabetes Investig. (2018) 9:173–8. doi: 10.1111/jdi.12655, PMID: 28294558 PMC5754522

[7] Yuan S Xu F Li X Chen J Zheng J Mantzoros CS. Plasma proteins and onset of type 2 diabetes and diabetic complications: Proteome-wide Mendelian randomization and colocalization analyses. Cell Rep Med. (2023) 4:101174. doi: 10.1016/j.xcrm.2023.101174, PMID: 37652020 PMC10518626

[8] Grassi MA Tikhomirov A Ramalingam SS Below JE Cox NJ Nicolae DL. Genome-wide meta-analysis for severe diabetic retinopathy. Hum Mol Genet. (2019) 28:2251–64. doi: 10.1093/hmg/ddr121, PMID: 21441570 PMC3098732

[9] Smith GD Hemani G. Mendelian randomization: genetic anchors for causal inference in epidemiological studies. Hum Mol Genet. (2014) 23:R89–98. doi: 10.1093/hmg/ddu328, PMID: 25064373 PMC4170722

[10] Csősz É Deák E Kalló G Csutak A Tőzsér J. Diabetic retinopathy: Proteomic approaches to help the differential diagnosis and to understand the underlying molecular mechanisms. J Proteom. (2017) 150:351–8. doi: 10.1016/j.jprot.2016.06.034, PMID: 27373871

[11] Yurekli BS Kocabas GU Aksit M Kutbay NO Suner A Yurekli I. The low levels of bone morphogenic protein-4 and its antagonist noggin in type 2 diabetes. Hormones (Athens). (2018) 17:247–53. doi: 10.1007/s42000-018-0041-5, PMID: 29943307

[12] Darwish NHE Hussein KA Elmasry K Ibrahim AS Humble J Moustafa M. Bone morphogenetic protein-4 impairs retinal endothelial cell barrier, a potential role in diabetic retinopathy. Cells. (2023) 12:1279. doi: 10.3390/cells12091279, PMID: 37174679 PMC10177364

[13] Al-Shabrawey M Hussein K Wang F Wan M Elmasry K Elsherbiny N. Bone morphogenetic protein-2 induces non-canonical inflammatory and oxidative pathways in human retinal endothelial cells. Front Immunol. (2021) 11:568795. doi: 10.3389/fimmu.2020.568795, PMID: 33584642 PMC7878387

[14] Abhary S Hewitt AW Burdon KP Craig JE. A systematic meta-analysis of genetic association studies for diabetic retinopathy. Diabetes. (2009) 58:2137–47. doi: 10.2337/db09-0059, PMID: 19587357 PMC2731535

[15] Krause C Guzman A Knaus P. Noggin. Int J Biochem Cell Biol. (2011) 43:478–81. doi: 10.1016/j.biocel.2011.01.007, PMID: 21256973

[16] Humble J Darwish N Elmasry K Moustafa M Awadalla F Ibrahim A. Bone Morphogenic Protein 4 (BMP4); a potential player in diabetic retinopathy[J. Invest Ophthalmol Visual Sci. (2023) 64:937–7.

[17] Al-Shabrawey M Hussein K Wang F Wan M Elmasry K Elsherbiny N. Bone morphogenetic protein-2 induces non-canonical inflammatory and oxidative pathways in human retinal endothelial cells[J. Front Immunol. (2021) 11:568795. doi: 10.3389/fimmu.2020.568795, PMID: 33584642 PMC7878387

[18] Chen Y Schlotterer A Lin J Dietrich N Fleming T Lanzinger S. Sex differences in the development of experimental diabetic retinopathy. Sci Rep. (2024) 14:22812. doi: 10.1038/s41598-024-73279-x, PMID: 39354039 PMC11445250

[19] Finan C Gaulton A Kruger FA Lumbers RT Shah T Engmann J. The druggable genome and support for target identification and validation in drug development. Sci Transl Med. (2017) 9(383):eaag1166. doi: 10.1126/scitranslmed.aag1166, PMID: 28356508 PMC6321762

[20] Zheng J Haberland V Baird D Walker V Haycock PC Hurle MR. Phenome-wide Mendelian randomization mapping the influence of the plasma proteome on complex diseases. Nat Genet. (2020) 52:1122–31. doi: 10.1038/s41588-020-0682-6, PMID: 32895551 PMC7610464

[21] Elsworth B Lyon M Alexander T Liu Y Matthews P Hallett J. The MRC IEU OpenGWAS data infrastructure. bioRxiv. (2020) 2020.08.10.244293. doi: 10.1101/2020.08.10.244293

[22] Zhu Z Zhang F Hu H Bakshi A Robinson MR Powell JE. Integration of summary data from GWAS and eQTL studies predicts complex trait gene targets. Nat Genet. (2016) 48:481–7. doi: 10.1038/ng.3538, PMID: 27019110

[23] Szklarczyk D Gable AL Lyon D Junge A Wyder S Huerta-Cepas J. STRING v11: protein-protein association networks with increased coverage, supporting functional discovery in genome-wide experimental datasets. Nucleic Acids Res. (2019) 47:D607–13. doi: 10.1093/nar/gky1131, PMID: 30476243 PMC6323986

[24] Wishart DS Feunang YD Guo AC Lo EJ Marcu A Grant JR. DrugBank 5.0: a major update to the DrugBank database for 2018. Nucleic Acids Res. (2018) 46(D1):D1074–82. doi: 10.1093/nar/gkx1037, PMID: 29126136 PMC5753335

[25] Yoo M Shin J Kim J Ryall KA Lee K Lee S. DSigDB: drug signatures database for gene set analysis. Bioinformatics. (2015) 31:3069–71. doi: 10.1093/bioinformatics/btv313, PMID: 25990557 PMC4668778

[26] Ferkingstad E Sulem P Atlason BA Sveinbjornsson G Magnusson MI Styrmisdottir EL. Large-scale integration of the plasma proteome with genetics and disease. Nat Genet. (2021) 53:1712–21. doi: 10.1038/s41588-021-00978-w, PMID: 34857953

[27] Robin X Turck N Hainard A Tiberti N Lisacek F Sanchez JC. pROC: an open-source package for R and S+ to analyze and compare ROC curves. BMC Bioinf. (2011) 12:77. doi: 10.1186/1471-2105-12-77, PMID: 21414208 PMC3068975

[28] Xiao B Liu L Li A Xiang C Wang P Li H. Identification and verification of immune-related gene prognostic signature based on ssGSEA for osteosarcoma. Front Oncol. (2020) 10:607622. doi: 10.3389/fonc.2020.607622, PMID: 33384961 PMC7771722

